# Supplementary material for: Genomic organization, domain assortments, and nucleotide-binding domain diversity of NLR proteins in Sordariales fungi
Source: PLoS Genet. 2025 Jul 7;21(7):e1011739. doi: 10.1371/journal.pgen.1011739 (PMC12258605; doi:10.1371/journal.pgen.1011739)
Supplement: S3 Fig — Branches corresponding to NLR sequences containing a Pkinase domain are highlighted in blue (Podospora bulbillosa), orange (Corynascus sp.), or red (other Chaetomiaceae species). Phylogeny was inferred using RAXML-NG on NACHT sequences aligned using MUSCLE. Nodes with a bootstrap value higher than 60% are highlighted with a black dot. (DOCX) [file pgen.1011739.s003.docx]

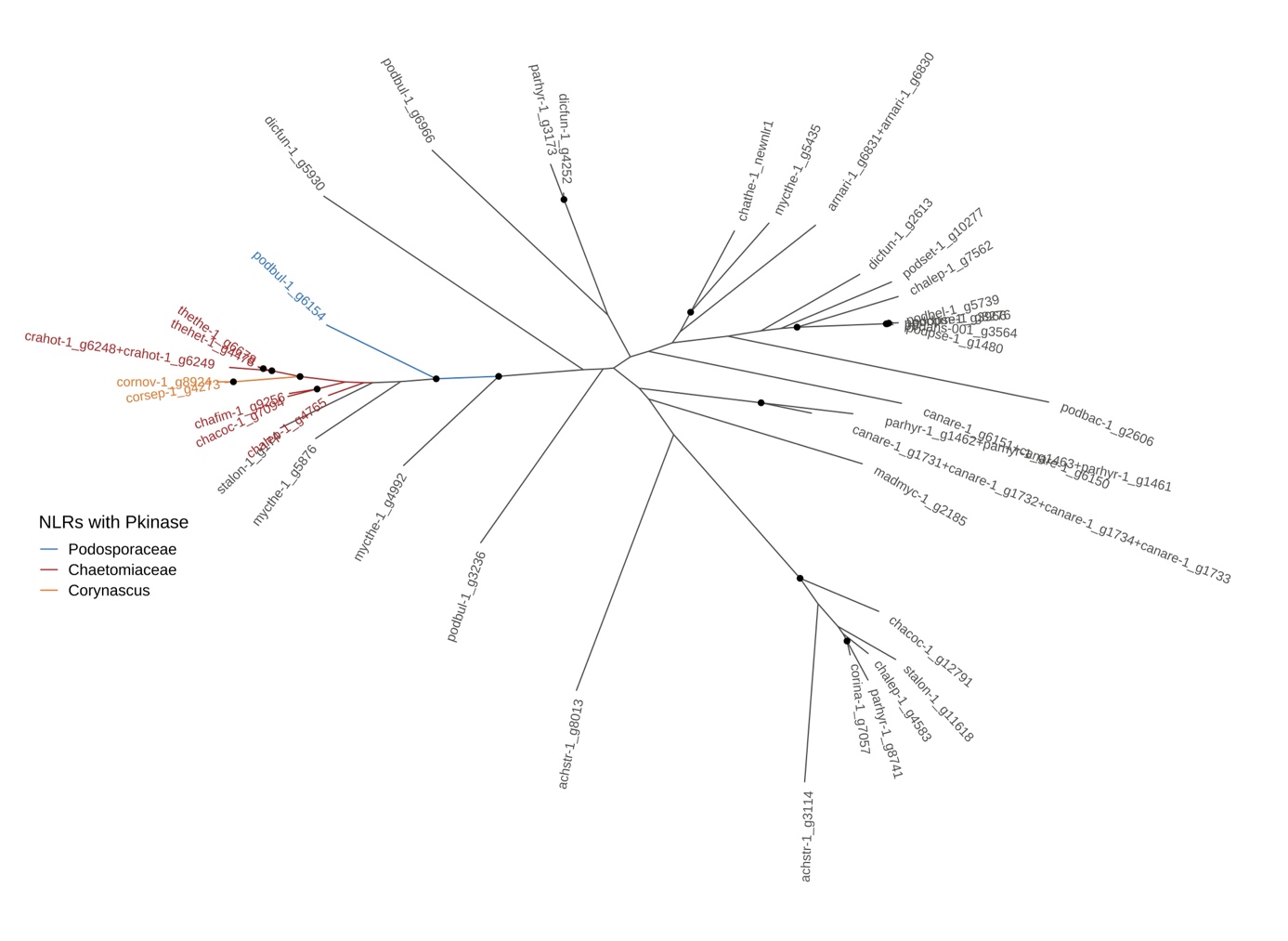


Figure S3. Phylogeny of all NACHT domains in orthogroups OGpodoTNACHT005 and OGchaetoTNACHT008. Branches corresponding to NLR sequences containing a Pkinase domain are highlighted in blue (Podospora bulbillosa), orange (Corynascus sp.), or red (other Chaetomiaceae species). Phylogeny was inferred using RAXML-NG on NACHT sequences aligned using MUSCLE. Nodes with a bootstrap value higher than 60% are highlighted with a black dot.
